# Blood and urinary metabolomic evidence validating traditional Chinese medicine diagnostic classification of major depressive disorder

**DOI:** 10.1186/s13020-018-0211-z

**Published:** 2018-10-25

**Authors:** Lan-Ying Liu, Hong-Jian Zhang, Li-Yuan Luo, Jin-Bao Pu, Wei-Qing Liang, Chun-Qin Zhu, Ya-Ping Li, Pei-Rong Wang, Yuan-Yuan Zhang, Chun-Yu Yang, Zhang-Jin Zhang

**Affiliations:** 1 Department of Psychiatry, Tongde Hospital of Zhejiang Province, Hangzhou, 310012 Zhejiang China; 2 Zhejiang Academy of Traditional Chinese Medicine, Hangzhou, 310007 Zhejiang China; 3 Department of Internal Chinese Medicine, Tongde Hospital of Zhejiang Province, Hangzhou, 310012 Zhejiang China; 4 School of Chinese Medicine, LKS Faculty of Medicine, The University of Hong Kong, 10 Sassoon Road, Pokfulam, Hong Kong, China

**Keywords:** Major depressive disorder, Traditional Chinese medicine, Classification, Metabolomics

## Abstract

**Background:**

Major depressive disorder (MDD) is a highly heterogeneous disease. Further classification may characterize its heterogeneity. The purpose of this study was to examine whether metabolomic variables could differentiate traditional Chinese medicine (TCM) diagnostic subtypes of MDD.

**Methods:**

Fifty medication-free patients who were experiencing a recurrent depressive episode were classified into Liver Qi Stagnation (LQS, n = 30) and Heart and Spleen Deficiency (HSD, n = 20) subtypes according to TCM diagnosis. Healthy volunteers (n = 28) were included as controls. Gas chromatography-mass spectrometry (GC–MS) was used to examine serum and urinary metabolomic profiles.

**Results:**

Twenty-eight metabolites were identified for good separations between TCM subtypes and healthy controls in serum samples. Both TCM subtypes had similar profiles in proteinogenic branched-chain amino acids (BCAAs) (valine, leucine, and isoleucine) and energy metabolism-related metabolites that were differentiated from healthy controls. The LQS subtype additionally differed from healthy controls in multiple amino acid metabolites that are involved in biosynthesis of monoamine and amino acid neurotransmitters, including phenylalanine, 3-hydroxybutric acid, *o*-tyrosine, glycine, l-tryptophan, and *N*-acetyl-l-aspartic acid. Threonic acid, methionine, stearic acid, and isobutyric acid are differentially associated with the two subtypes.

**Conclusions:**

While both TCM subtypes are associated with aberrant BCAA and energy metabolism, the LQS subtype may represent an MDD subpopulation characterized by abnormalities in the biosynthesis of monoamine and amino acid neurotransmitters and closer associations with stress-related pathophysiology. The metabolites differentially associated with the two subtypes are promising biomarkers for predicting TCM subtype-specific antidepressant response [registered at http://www.clinicaltrials.gov (NCT02346682) on January 27, 2015].

**Electronic supplementary material:**

The online version of this article (10.1186/s13020-018-0211-z) contains supplementary material, which is available to authorized users.

## Background

Major depressive disorder (MDD) is a highly heterogeneous mental illness with a wide range of clinical manifestations and multi-system etiopathogenesis. The core symptoms of low mood and loss of interest and pleasure are often accompanied by somatic and other psychiatric symptoms, such as pain, sleep disturbance, anxiety, and neurocognitive dysfunction [1]. Although a great attempt has been made to characterize its heterogeneity by subdividing MDD based on its clinical features, severity, and polygenic features, convincing evidence for the existence of depressive symptom dimensions and symptomatic subtypes is lacking, mainly due to symptomatic diversity and the absence of patterns [2–5]. Therefore, a novel classification system that could improve clinical applicability and more precisely identify pathological heterogeneity of MDD is highly desirable.

As an ancient medical practice, traditional Chinese medicine (TCM) has an established distinct diagnostic system. One key approach to diagnosis is the differentiation of etiopathological pattern which is generally defined by a comprehensive analysis of clinical symptoms and signs collected through inspection, auscultation, olfaction, interrogation, and palpation of the pulses [6]. Over the past decade, several large-scale studies have revealed 12 different TCM patterns of MDD on the basis of the modern psychiatric diagnostic instruments and analysis tools, such as latent tree model analysis [7–12]. Despite disagreements on some patterns, a consensus on clinical diagnostic criteria for the two most common and opposing patterns, Liver Qi Stagnation (LQS) and Heart and Spleen Deficiency (HSD), which account for approximately 2/3 of depressed patients, have been reached as shown in Table 1 [7, 9, 10].

**Table 1:** Clinical manifestations and diagnostic criteria of TCM-based subtypes of MDD

	Liver Qi Stagnation (LQS)	Heart and Spleen Deficiency (HSD)
Mood symptom characters	Depressed mood with frustration, nervousness, and/or irritability	Depressed mood with excessive pensiveness, suspicion, and/or timorousness
Somatic symptoms	A. Frequently sighing (0 = absent, 1 = slight, 2 = mild, 3 = moderate, 4 = severe) B. Chest distension and/or hypochondriac pain (0 = absent, 1 = slight, 2 = mild, 3 = moderate, 4 = severe) C. Abdominal bloating (0 = absent, 1 = slight, 2 = mild, 3 = moderate, 4 = severe) D. Decreased appetite (0 = absent, 1 = mild, 2 = moderate, 3 = severe) E. Loose stool (0 = absent, 1 = mild, 2 = moderate, 3 = severe) F. Breast tenderness (0 = absent, 1 = slight, 2 = mild, 3 = moderate, 4 = severe) G. Irregular menstruation (0 = normal, 1 = seldom, 2 = sometimes, 3 = frequent)^a^ H. Menstrual pain (0 = absent, 1 = mild, 2 = moderate, 3 = severe)^a^	A. Palpitation (0 = normal, 1 = seldom, 2 = sometimes, 3 = most times, 4 = all times) B. Forgetfulness (0 = absent, 1 = slight, 2 = mild, 3 = moderate, 4 = severe) C. Insomnia or dream-disturbed sleep (0 = absent, 1 = sometimes, 3 = most times) D. Decreased appetite (0 = absent, 1 = slight, 2 = mild, 3 = moderate, 4 = severe) E. Abdominal fullness (0 = absent, 1 = sometimes, 3 = most times) F. Loose stool or dysfunctional diarrhea (0 = absent, 1 = slight, 2 = mild, 3 = moderate, 4 = severe) G. Pale and sallow complexion (0 = normal, 1 = slightly apparent, 2 = mildly apparent; 3 = moderately apparent, 4 = very apparent) H. Tiredness (0 = absent, 1 = mild, 2 = moderate, 3 = severe)
Tongue and pulse	I. Red tongue body with thin and white coating (0 = normal, 1 = mildly apparent, 2 = moderately apparent, 3 = very apparent) J. Wiry pulse (0 = normal, 1 = slightly apparent, 2 = mildly apparent; 3 = moderately apparent, 4 = very apparent)	I. Pale and tender or watery tongue body with white coating (0 = normal, 1 = slightly apparent, 2 = mildly apparent; 3 = moderately apparent, 4 = very apparent) J. Weak and thin pulse (0 = normal, 1 = slightly apparent, 2 = mildly apparent; 3 = moderately apparent, 4 = very apparent)
Diagnostic criteria^b^	[1]. Must have A, B, I, and J; at least one of D and E; and at least one of F, G, and H for women [2]. Total score of physical symptoms and tongue and pulse signs is not less than 10	[1]. Must have at least two of A, B and C; at least two of D, E and F; at least one of G and H; I and J [2]. Total score of physical symptoms and tongue and pulse signs is not less than 10

The diagnostic criteria are modified based on Ref. [7, 9, 10]

^a^ F, G, and H items are only applied for women

^b^ Those who fail to meet either LQS or HSD subtype are classified as Other Subtypes

Differential profiles of functional brain connectivity has been observed between “deficiency” and “excessive” patterns of MDD [13]. One recent study has further revealed differences between patients with liver-depression and spleen-deficiency syndrome and healthy volunteers in multiple serum metabolic pathways associated with amino acid and energy metabolism [14]. On the other hand, aberrant amino acid metabolism that is involved in protein synthesis, energy metabolism, monoamine and amino acid neurotransmitter biosynthesis has been suggested in the pathogenesis of MDD and antidepressant response [15]. Differential metabolomic profiles are also associated with the severity of MDD and whether the patients had early life stress and suicidal ideation [16–20]. These studies have led to the hypothesis that certain metabolic pathways particularly associated with protein synthesis, energy metabolism, and biosynthesis of monoamine and amino acid neurotransmitters could serve valid biomarkers for differentiating TCM diagnostic subtypes of MDD.

To test this hypothesis, this study was designed to examine whether blood and urinary metabolomic profiles were different between the LQS and HSD subtypes of MDD with the inclusion of healthy volunteers as controls.

## Materials and methods

### Setting and participants

This study was conducted at Tongde Hospital of Zhejiang Province in Hangzhou, China between April 2015 and March 2017. The study protocol was approved by the Medical Ethical Committee [equivalent to the Institutional Review Board (IRB)] of Tongde Hospital of Zhejiang Province on March 11, 2014 (The approved document is attached in Additional file 1) and registered at http://www.clinicaltrials.gov (NCT02346682) on January 27, 2015 (https://clinicaltrials.gov/ct2/show/NCT02346682). All participants gave voluntary, written, informed consent before entering the study. We reported this study according to the Minimum Standards of Reporting Checklist (see Additional file 2).

**Fig. 1:**
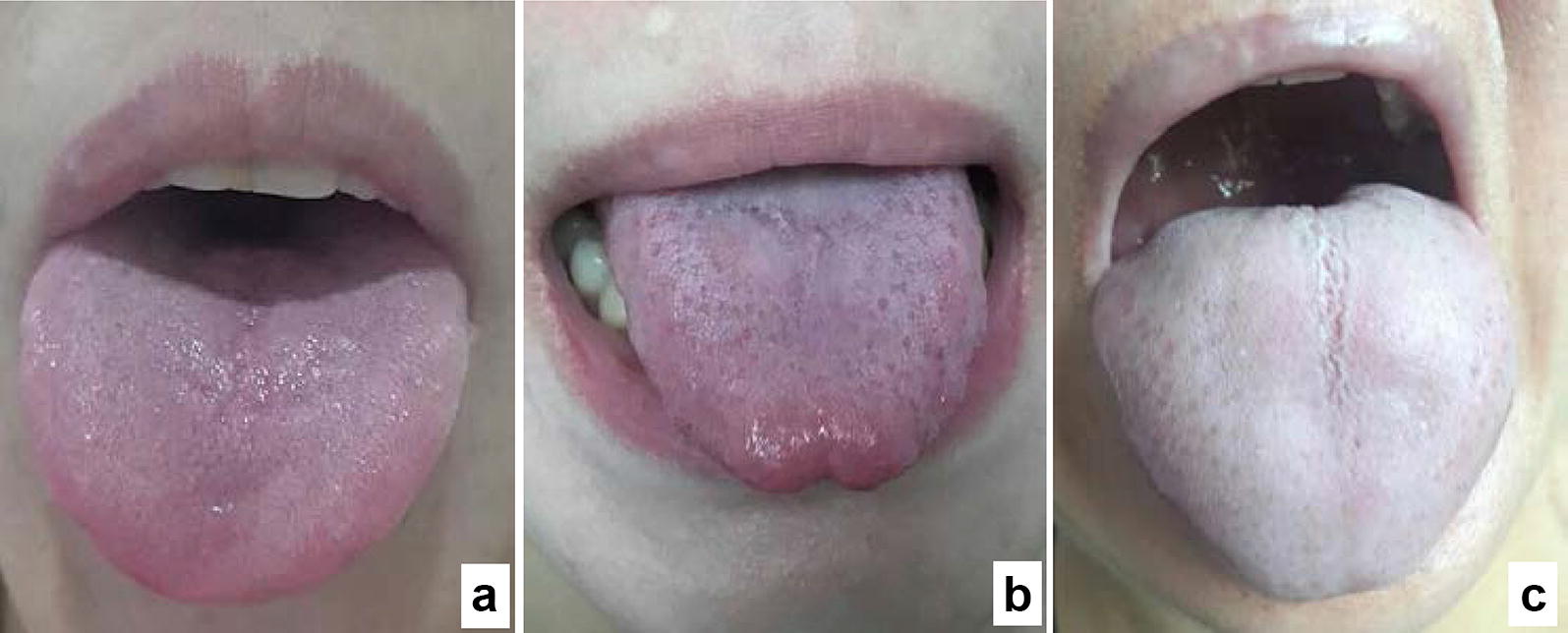
Representative tongue pictures taken from a healthy volunteer (**a**), depressed patients with Liver Qi Stagnation (**b**) and Heart Spleen Deficiency (**c**) subtypes

Participants were sought from out-inpatients. Subjects were eligible for this study if they: (a) were aged 18–65 years; (b) were currently experiencing a recurrent, moderate or severe depressive episode according to the Diagnostic and Statistical Manual of Mental Disorders, Fifth Edition (DSM-5), as evidenced by a score of at least 21 on the 24-item Hamilton Rating Scale for Depression (HAMD-24) [21]; (c) met the diagnostic criteria of the LQS or HSD subtype as defined in Table 1 with representative tongue manifestations as shown in Fig. 1; and (d) had received no treatment with antidepressants and other psychotropic drugs in the previous 3 months. Tongue diagnosis is a critical diagnostic approach in discriminating different TCM patterns according to manifestations of the tongue coating and body [22].

Patients were excluded from this study if they: (a) were experiencing their first episode of depression; (b) had serious comorbid cardiac, hepatic, or renal conditions; (c) had a history of brain injury or surgery; (d) had a history of manic, hypomanic, or mixed episodes; (e) had received investigational drug treatment within the previous 6 months; (f) had experienced alcohol or drug abuse within the previous 12 months; or (g) were pregnant or breastfeeding.

A group of healthy volunteers who had no personal or family history of significant mental and physical illness was recruited from Tongde Hospital and local community to serve as controls.

### Screening, assessment, and TCM diagnosis

The screening of patients and healthy volunteers was done by a psychiatrist and a TCM practitioner. The severity of the depression symptoms of the patients and healthy controls was assessed using HAMD-24 [21]. The assessment was conducted by a trained rater (L.Y.L., C.Q.Z.). TCM subtypes diagnoses were made by at least two senior TCM practitioners (Y.P.L., C.Q.Z.) and a third TCM practitioner (L.Y.L.) was involved in the diagnosis process if the first two practitioners could not reach an agreement.

To ensure consistency of assessment and diagnosis, a manual was provided and a training workshop was carried out on videotaped patients with different TCM subtypes before the study was initiated. An inter-rater reliability coefficient of > 0.85 was achieved after the completion of the training workshop.

### Collection and preparation of serum and urine samples

Blood and urine sample for each were collected within 2 days after the completion of clinical assessment. Following the sample collection, patients immediately received antidepressant and other psychotropic treatment at psychiatrists’ discretion based on their clinical condition, with routine subsequent monitoring.

In the morning (09:00–10:00) after an overnight fasting, 10 ml of midstream urine were collected; 15 ml of blood were drawn and sera were immediately separated. Samples were further prepared for metabolomic measurement as described previously [17, 23]. Briefly, 500 μl of urine or sera was mixed with 50 μl heptanoic acid and 10 mg/ml para-chlorophenylalanine as the internal standard solution. 500 μl methanol was added to precipitate protein, mixed for 5 min, and then centrifuged at 12,000 rpm/min for 15 min at 4 °C. A 500-μl aliquot of supernatant was transferred to a clean Eppendorf tube and dried under a low temperature vacuum drier. The residue was derivatized by adding 100 μl methoxyamine hydrochloride (15 mg/ml in pyridine) at 30 °C for 90 min. For each sample, 100 μl BSTFA (1% TMCS) was added and the mixture was heated at 70 °C for 60 min. The derivative was cooled and filtered in 0.45 μm membrane prior to GC–MS analysis. Quality control samples and the reference standard were processed as done for experimental samples.

### GC–MS acquisition

One μl aliquot of derivatized sample was injected into a Varian 450-GC/240-MS equipped with 19091 N-113 capillary column (30 m × 0.32 mm × 0.25 μm, Agilent J & W Scientific, USA) at a split ratio of 10:1. Helium was used as the carrier gas with a constant flow rate of 1 ml/min. The initial temperature was set at 70 °C for 4 min, elevated to 300 °C at a rate of 8°C/min, and then maintained for 3 min. Temperature for the injector, transfer line, and ion source was set at 280 °C, 250 °C, and 220 °C, respectively. The mass range (50–800 m/z) in a full-scan mode for electron impact ionization (1.0 kV) was applied. The solvent delay time was set to 6 min.

### Statistical analysis

There were no studies detecting metabolomic effects in TCM subtypes of MDD. Sample size estimation was based on one previous study that has revealed differential metabolomic profiles between MDD patients with and without early life stress [17]. As the LQS and HSD subtypes are appear to be differentially associated with stress-related (reactive) and endogenous (melancholic) depression [2, 9], we assumed that metabolomic differences between the two TCM subtypes was similar to that of MDD patients with and without early life stress [17]. The study has shown an averaged 36% difference in plasma level of major metabolites between MDD patients with and without early life stress with an averaged standard deviation of 41% [17]. A sample size of 22 each group would be sufficient to yield an 80% power at a statistical level of 0.05.

For baseline data, one-way analysis of variance (ANOVA) was used to detect differences in continuous variables among healthy controls and the two TCM subtypes. Student’s *t*-test was used to detect differences in continuous variables between the two TCM subtypes. Categorical baseline variables were analyzed using Chi square (χ^2^) test.

For metabolomic data, the pretreatment process was performed, including novel nonlinear retention time alignment, baseline filtration, peak identification, matching, and integration. The resulting data matrix consisting of variables, sample code, and peak area was further processed using Microsoft Excel program. The original spectral data obtained from GC-MS spectroscopy were scaled to unit variance (*z*), which was calculated from the formula *z* = (*x* – *y*)/*s*, where *x*, *y*, and *s* represent the level of the particular metabolite in one subject, the mean level and the standard deviation of this metabolite across all subjects, respectively.

Nonparametric Mann–Whitney U test was used to detect significantly differential metabolites. All metabolites were determined by standard samples and/or a similarity of > 70% that was obtained by comparing with the mass spectral database of the US National Institute for Standards and Technology (NIST).

Metabolites were determined using variable importance in the projection (VIP) which value was defined as > 1 and *t*-test was set at a level of 95%. The principal component analysis (PCA) was used to discriminate metabolic patterns among the three groups. The orthogonal projection to latent structures-discriminant analysis (OPLS-DA) model was further constructed to identify meaningful metabolites that could differentiate between the three groups using SIMCA-P 13.0 program. A regression method was applied to establish the optimal discriminant model with controlling gender as a confounding factor. The quality of the model was tested with cross-validation and R^2^X, R^2^Y, and Q^2^ values were obtained. OPLS-DA models which all R^2^X, R^2^Y, and Q^2^ values were ≥ 0.5 were acceptable. Hierarchical clustering patterns were further established with heatmap analysis using R i386 3.3.0 software for visual verification of OPLS-DA models. The impact pathway was determined using MetaboAnalyst 3.0 (http://www.metaboanalyst.ca), a web-based tool for pathway analysis, with the criteria as *p*-value < 0.05, false discovery rate (FDR) < 0.05, and impact value > 0. Visualization of metabolomic correlation network was drawn with CytoScape 3.3.0. The receiver operating characteristic (ROC) curve analysis was conducted to determine the optimal metabolite combination patterns that could well dichotomize the subtypes and healthy controls at acceptable sensitivity and specificity (defined as greater than 80% for both).

Statistical analysis was conducted using SPSS 18.0 (SPSS Inc., Chicago, IL, USA) and, unless otherwise indicated, significance level was set at a two-tailed *P* < 0.05.

## Results

### Participant characteristics

**Fig. 2:**
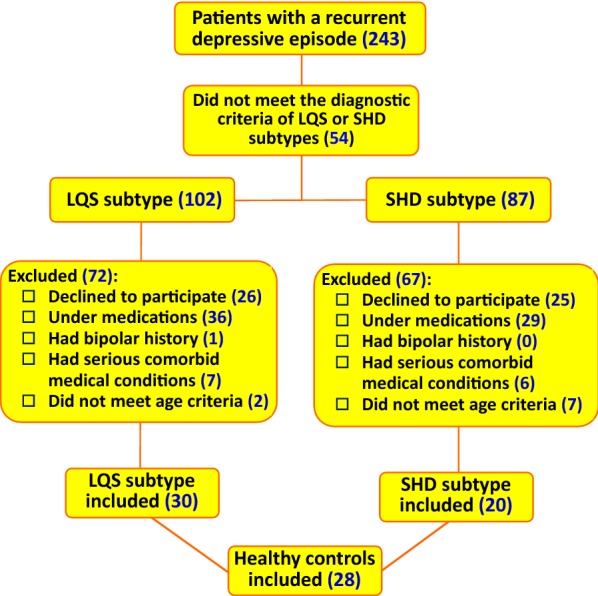
Flowchart of screening and recruitment. *LQS* Liver Qi Stagnation, *HSD* Heart and Spleen Deficiency

Flowchart of screening and recruitment is shown in Fig. 2. Of 243 depressed patients screened, 189 met the diagnostic criteria of LQS (n = 102) and SHD (n = 87) subtype; 27.0% (51/189) rejected to participate the study; 30 LQS subtypes and 20 SHD subtypes who met the inclusion criteria but did not meet any exclusion criteria were recruited. Twenty-eight healthy volunteers were recruited to serve as controls. The proportion of female participants of the LQS subtype was significantly higher than that of the HSD subtype (χ^2^ = 6.696, p = 0.035) (Table 2). Other demographic and clinical variables were not different among the three groups.

**Table 2:** Demographic and clinical characteristics of participants

Variables	Healthy controls (n = 28)	LQS subtype^a^ (n = 30)	HSD subtype^a^ (n = 20)	*p* value^b,c^
Female, n (%)^b^	17 (60.7)	25 (83.3)	10 (50.0)	0.035
Age (y)^c^	34.1 ± 8.1	38.1 ± 13.4	41.5 ± 13.7	0.107
Educational level, n (%)^b^				0.331
Primary	2 (7.1)	6 (20.0)	5 (25.0)	
Middle	5 (17.9)	4 (13.3)	5 (25.0)	
High^d^	21 (75.0)	20 (66.7)	10 (50.0)	
Marital status, n (%)^b^				0.734
Married	23 (82.1)	26 (86.7)	18 (90.0)	
Single/divorced/widowed	5 (17.9)	4 (13.3)	2 (10.0)	
Income level, n (%)^b,e^				0.775
Low	4 (14.3)	7 (23.3)	3 (15.0)	
Middle	17 (60.7)	18 (60.0)	14 (70.0)	
High	7 (25.0)	5 (16.7)	3 (15.0)	
Family history with severe mental diseases, n (%)^b,f^	3 (10.7)	4 (13.3)	0	0.250
Comorbid conditions, n (%)^b,g^		3 (10)	4 (20)	0.560
Medication history, n (%)^b,h^				0.803
SSRIs/SNRIs^a^		25 (83.3)	18 (90.0)	
Antipsychotics		5 (16.7)	2 (10.0)	
Previous depressive episode, n (%)^b^				0.684
Single		18 (60.0)	10 (50.0)	
Multiple		12 (40.0)	10 (50.0)	
Duration of the illness (months)^c,i^		39.5 ± 60.9	33.6 ± 47.8	0.716
HAMD-24 score^a,c^		32.5 ± 8.5	30.1 ± 6.5	0.286

^a^ *LQS* Liver Qi Stagnation, *HSD* Heart and Spleen Deficiency, *SSRIs* selective serotonin reuptake inhibitors, *SNRIs* serotonin–norepinephrine reuptake inhibitors, *HAMD-24* 24-item Hamilton Rating Scale for Depression

^b^ Categorical data was analyzed using Chi square (χ^2^) test

^c^ Continuous data are expressed mean ± SD and analyzed using one-way analysis of variance (ANOVA) for the three groups and Student t-test for LQS and HSD subtypes

^d^ High levels included high school, college, and postgraduate education

^e^ Compared to average local household incomes

^f^ Severe mental diseases mainly included schizophrenia, bipolar disorders, and severe depression

^g^ Most comorbid conditions were cardiovascular and diabetic diseases

^h^ SSRIs mainly included paroxetine, sertraline, citalopram, and fluoxetine. SNRIs mainly include venlafaxine and duloxetine. Antipsychotics mainly include quetiapine, olanzapine, and risperidone. All medications were taken earlier than 3 months ago at screening

^i^ Duration of the illness was calculated from the first episode of depression

### Clustering patterns of differential metabolite profiles

**Fig. 3:**
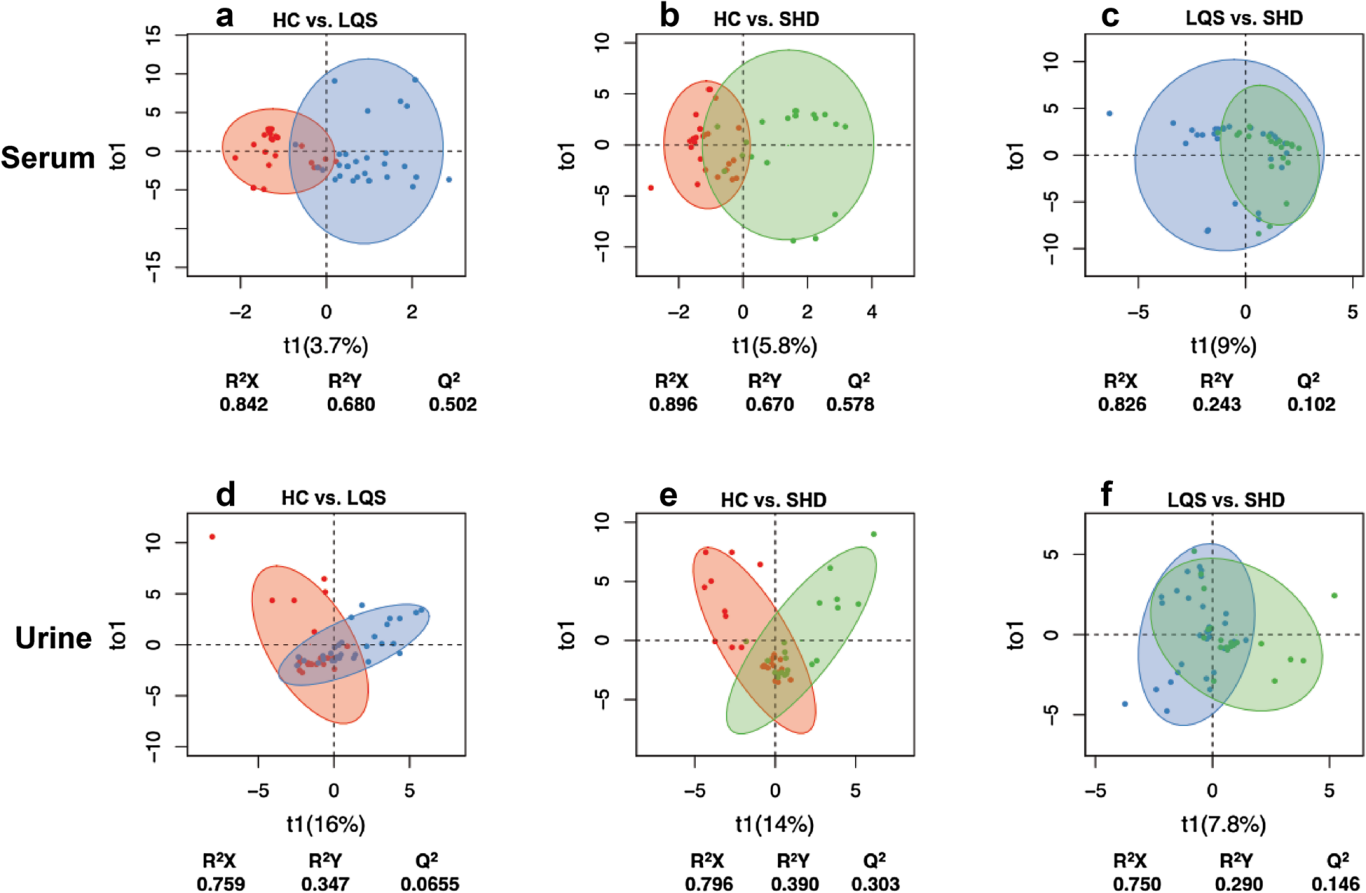
Clustering analysis of serum (**a**, **b**, **c**) and urine (**d**, **e**, **f**) metabolomic profiles. OPLS-DA models were built in patients with Liver Qi Stagnation (LQS) and Heart and Spleen Deficiency (HSD) subtypes of MDD and healthy controls (HC). Acceptable criteria for the models were defined as all R^2^X, R^2^Y, and Q^2^ values of ≥ 0.5. Comparisons were conducted between HC and LQS (**a**, **d**), HC and HSD (**b**, **e**), and LQS and HSD (**c**, **f**)

**Fig. 4:**
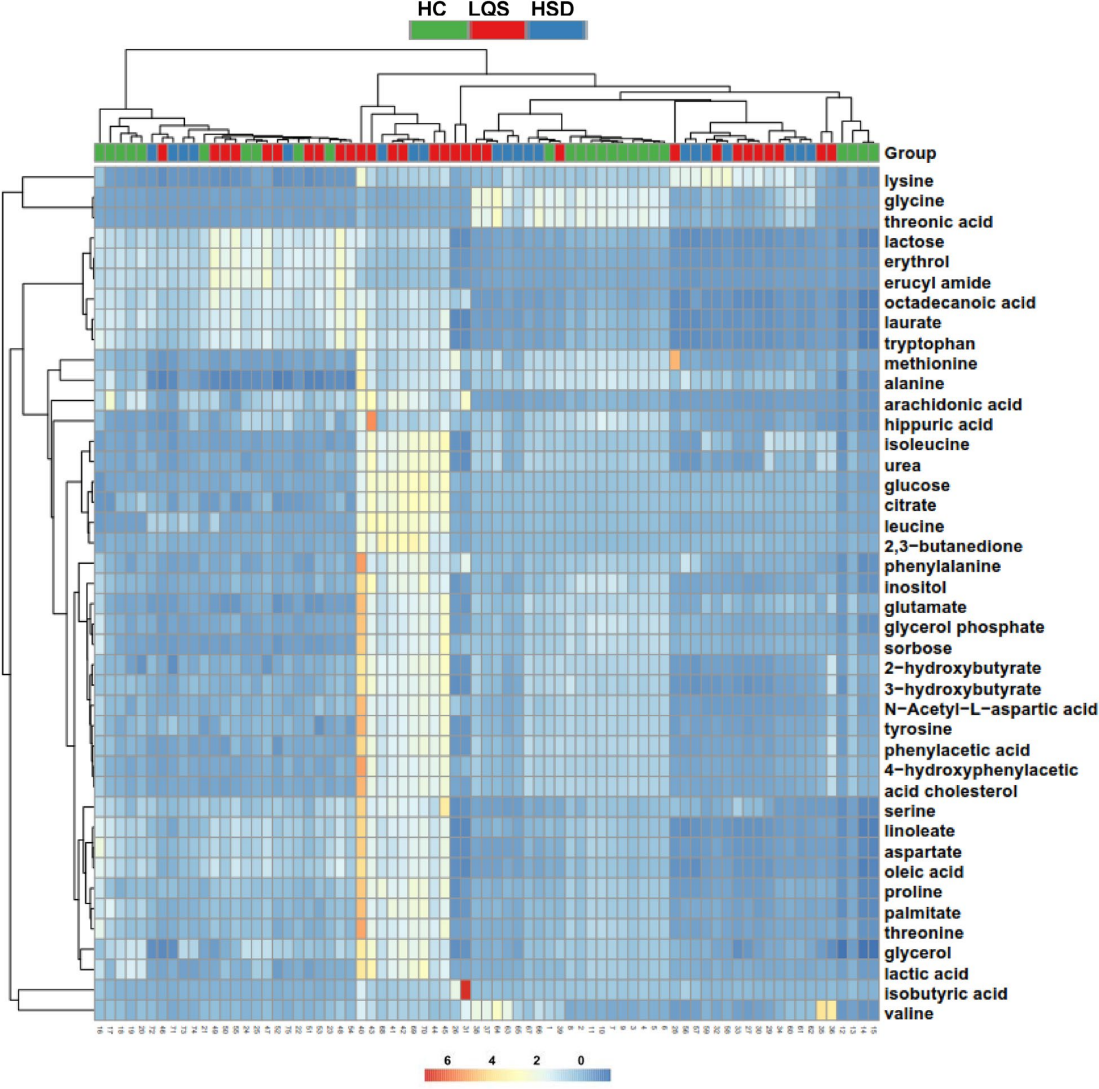
Heat maps generated from hierarchical Pearson clustering show metabolites in serum samples obtained from Liver Qi Stagnation (LQS) and Heart and Spleen Deficiency (HSD) subtypes of MDD and healthy controls (HC)

**Fig. 5:**
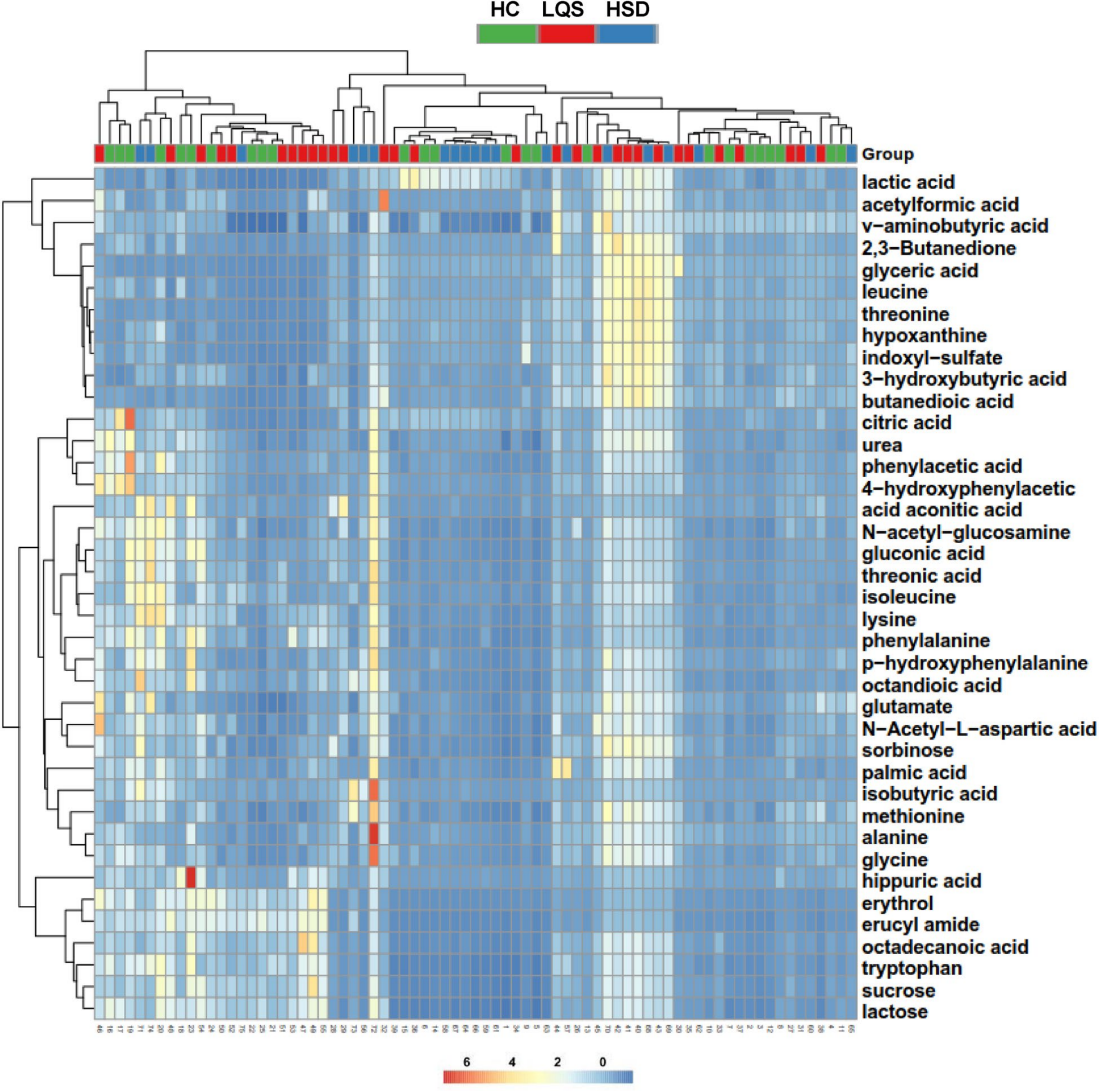
Heat maps generated from hierarchical Pearson clustering show metabolites in urine samples obtained from Liver Qi Stagnation (LQS) and Heart and Spleen Deficiency (HSD) subtypes of MDD and healthy controls (HC)

A total of 28 metabolites were identified to well differentiate the two TCM subtypes from healthy controls (Table 3); 8 of them were present in both serum and urine samples and/or in the two subtypes. The OPLS-DA models revealed that serum metabolite profiles of both subtypes were clearly separated from healthy controls with R^2^X values of ≥ 0.842, R^2^Y values of ≥ 0.670, and Q^2^ values of ≥ 0.502 (Fig. 3a, b). Urinary metabolite profiles of the two subtypes were not well separated from healthy controls, with considerable overlaps and low R^2^Y and Q^2^ values (Fig. 2d, e). The two subtypes showed large overlaps in serum and urinary metabolite profiles with low R^2^Y and Q^2^ values (Fig. 2c, f). Hierarchical clustering analysis displayed remarkable differences in mentalism between the three groups, visually validating OPLS-DA models in serum and urine samples (Figs. 4 and 5).

**Table 3:** Differential metabolites identified from MDD patients with TCM subtypes and healthy controls ^a^

Group samples	Metabolites	Formula	*r* ^b^	*p*-value^c^	FC (MDD/HC)^a^	Pathway
*Serum: LQS vs. HC* ^a^						
1^d^	l-Valine	C_5_H_11_NO_2_	0.236	0.009	0.930	Aminoacyl-tRNA biosynthesis
2^d^	l-Phenylalanine	C_9_H_11_NO_2_	0.199	0.237	0.350	Aminoacyl-tRNA biosynthesis
3^d^	l-Lysine	C_6_H_14_N_2_O_2_	0.150	0.761	0.459	Aminoacyl-tRNA biosynthesis
4	l-Proline	C_5_H_9_NO_2_	0.143	0.447	0.182	Aminoacyl-tRNA biosynthesis
5^d^	l-Leucine	C_6_H_13_NO_2_	0.124	0.000	1.047	Aminoacyl-tRNA biosynthesis
6^e^	Isobutyric acid	C_4_H_8_O_2_	0.103	0.565	0.702	Protein digestion and absorption
7	l-Lactic acid	C_3_H_6_O_3_	− 0.147	0.370	0.013	Propanoate metabolism
8	Glycine	C_2_H_5_NO_2_	− 0.151	0.279	− 0.954	Aminoacyl-tRNA biosynthesis
9	Threonic acid	C_4_H_8_O_5_	− 0.175	0.250	− 1.080	Ascorbate and aldarate metabolism
*Urine: LQS vs. HC* ^a^						
1	l-Sorbose	C_6_H_12_O_6_	0.149	0.624	0.661	Unknown
2	Diacetyl	C_4_H_6_O_2_	0.147	0.120	0.825	Butanoate metabolism
3	*N*-Acetyl-d-glucosamine	C_8_H_15_NO_6_	0.146	0.748	0.304	Amino sugar and nucleotide sugar metabolism
4^d^	l-Methionine	C_5_H_11_NO_2_S	0.144	0.417	0.546	Cysteine and methionine metabolism
5^d^	3-Hydroxybutyric acid	C_4_H_8_O_3_	0.139	0.088	0.819	Butanoate metabolism
6^d^	Pyruvic acid	C_3_H_4_O_3_	0.125	0.116	1.085	Citrate cycle (TCA cycle)
7^d^	Stearic acid	C_18_H_36_O_2_	0.117	0.327	0.490	Fatty acid biosynthesis
8	trans-Aconitic acid	C_6_H_6_O_6_	0.115	0.648	0.444	C5-Branched dibasic acid metabolism
9^d^	Glycine	C_2_H_5_NO_2_	0.111	0.685	0.441	Aminoacyl-tRNA biosynthesis
10^d^	*N*-Acetyl-l-aspartic acid	C_6_H_9_NO_5_	0.107	0.176	0.680	Alanine, aspartate and glutamate metabolism
11	Threonic acid	C_4_H_8_O_5_	0.101	0.906	0.428	Ascorbate and aldarate metabolism
12^d^	l-Tryptophan	C_11_H_12_N_2_O_2_	− 0.108	0.054	0.186	Glycine, serine and threonine metabolism
13	α-Lactose	C_12_H_22_O_11_	− 0.114	0.116	0.259	Galactose metabolism
14^d^	l-Lactic acid	C_3_H_6_O_3_	− 0.129	0.735	0.263	Propanoate metabolism
15^d^	Palmitic acid	C_16_H_32_O_2_	− 0.149	0.005	0.303	Fatty acid metabolism
16	Indoxyl sulfate	C_8_H_7_NO_4_S	− 0.164	0.673	0.583	Unknown
17^d^	*o*-Tyrosine	C_9_H_11_NO_3_	− 0.203	0.217	0.080	Unknown
18	*p*-Hydroxyphenylacetic acid	C_9_H_8_O_3_	− 0.122	0.673	− 0.120	Tyrosine metabolism
19^d^	Citric acid	C_6_H_8_O_7_	− 0.156	0.015	− 0.265	Citrate cycle (TCA cycle)
*Serum: HSD vs. HC* ^a^						
1^d^	l-Valine	C_5_H_11_NO_2_	0.226	0.079	0.689	Aminoacyl-tRNA biosynthesis
2^d^	l-Leucine	C_6_H_13_NO_2_	0.161	0.000	1.120	Aminoacyl-tRNA biosynthesis
3^d^	l-Lysine	C_6_H_14_N_2_O_2_	0.148	0.337	0.722	Aminoacyl-tRNA biosynthesis
4	l-Threonine	C_4_H_9_NO_3_	− 0.105	0.000	− 0.191	Glycine, serine and threonine metabolism
5	d-chiro-Inositol	C_6_H_12_O_6_	− 0.135	0.138	− 0.200	Inositol phosphate metabolism
6^d^	l-Methionine	C_5_H_11_NO_2_S	− 0.140	0.009	− 0.299	Cysteine and methionine metabolism
7	l-Lactic acid	C_3_H_6_O_3_	− 0.170	0.253	− 0.183	Propanoate metabolism
*Urine: HSD vs. HC* ^a^						
1^d^	l-Isoleucine	C_6_H_13_NO_2_	0.161	0.007	0.681	Valine, leucine and isoleucine degradation
2	Threonic acid	C_4_H_8_O_5_	0.113	0.349	0.687	Ascorbate and aldarate metabolism
3	*N*-Acetyl-d-glucosamine	C_8_H_15_NO_6_	0.109	0.283	0.432	Amino sugar and nucleotide sugar metabolism
4^d^	Stearic acid	C_18_H_36_O_2_	− 0.104	0.002	0.019	Fatty acid biosynthesis
5	α-Lactose	C_12_H_22_O_11_	− 0.116	0.000	− 0.126	Galactose metabolism
*Urine: LQS vs. HSD* ^a^						
1	Isobutyric acid	C_4_H_8_O_2_	0.127	0.304	1.114^e^	Protein digestion and absorption

^a^ *FC* fold change, *MDD* major depressive disorder, *HC* healthy controls, *LQS* Liver Qi Stagnation, *HSD* Heart and Spleen Deficiency

^b^ Correlation coefficients (*r*) were obtained from OPLC-DA with a threshold of 3.010. Positive and negative coefficients respectively indicate higher and lower levels of metabolites compared to healthy controls

^*c*^ *p*-values were obtained from Wilcoxon-Mann–Whitney test between TCM subtypes and healthy controls

^d^ Metabolites were determined using standard samples

^e^ FC value represents LQS/HSD

### Differential metabolites of TCM subtypes

#### Between LQS subtype and healthy controls

**Fig. 6:**
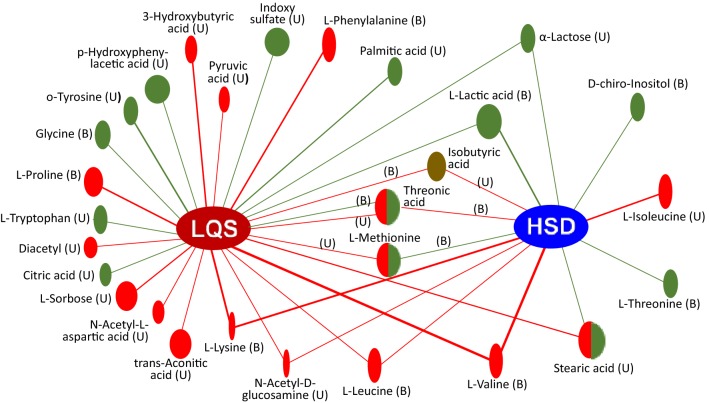
Correlation network analysis of 28 differential metabolites among Liver Qi Stagnation (LQS) and Heart and Spleen Deficiency (HSD) subtypes of MDD versus healthy controls. Green and red lines with ovals indicates negative and positive correlation, respectively. The ovals with half green and half red colors indicate mixed correlations. The oval size represents *p* value compared with healthy controls. Isobutyric acid (dark brown oral) has a significant difference between the two subtypes. *B* blood samples, *U* urine samples

24 metabolites were valid in discriminating the LQS subtype from healthy controls (Fig. 6, Table 3). In serum samples, levels of l-valine, l-phenylalanine, l-lysine, l-proline, l-leucine, and isobutyric acid were markedly higher than those of healthy controls and positively correlated with the LQS subtype. l-lactic acid level of the LQS subtype was greater than that of healthy subjects with negative correlation with the subtype. Levels of glycine and threonic acid were significantly lower than those of healthy controls and negatively correlated with the LQS subtype.

In urine samples, levels of l-sorbose, diacetyl, *N*-acetyl-d-glucosamine, l-methionine, 3-hydroxybutyric acid, pyruvic acid, stearic acid, trans-aconitic acid, glycine, *N*-acetyl-l-aspartic acid, and threonic acid were significantly higher than those of healthy subjects and positively correlated with the LQS subtype. Levels of l-tryptophan, α-lactose, l-lactic acid, palmitic acid, indoxyl sulfate, and o-tyrosine were significantly greater than those of healthy subjects but negatively correlated with the LQS subtype. Levels of *p*-hydroxyphenylacetic acid and citric acid were significantly lower higher than those of healthy subjects and negatively correlated with the LQS subtype.

#### Between HSD subtype and healthy controls

13 metabolites were identified to be effective in distinguishing the HSD subtype from healthy controls (Fig. 6, Table 3). In serum samples, levels of l-valine, l-leucine, and l-lysine were remarkably higher than those of healthy subjects and positively correlated with the HSD subtype. Levels of l-threonine, d-chiro-Inositol, l-methionine, and l-lactic acid were significantly lower than those of healthy subjects and negatively correlated with the HSD subtype.

In urine samples, levels of l-Isoleucine, isobutyric acid, threonic acid, and *N*-acetyl-d-glucosamine were considerably higher than those of healthy subjects and positively correlated with the HSD subtype. Level of stearic acid was also significantly higher than that of healthy controls but negatively correlated with the HSD subtype. Level of α-lactose was significantly lower than that of healthy controls and negatively correlated with the HSD subtype.

#### Between LQS and HSD subtypes

10 metabolites showed correlations with both subtypes (Fig. 6). They are urine α-lactose, *N*-acetyl-d-glucosamine, and stearic acid; serum l-lactic acid, l-lysine, l-valine, and l-leucine; and isobutyric acid, threonic acid, and l-methionine in both serum and urine samples. Most metabolite correlates with the two subtypes were consistent. Serum threonic acid correlate was negative with the LQS subtype but positive with the HSD subtype. Urine stearic acid correlate was positive with the LQS subtype but negative with the HSD subtype. l-Methionine correlate was positive with the LQS subtype in urine samples but negative with the HSD subtype in serum samples. The LQS subtype had a markedly lower urinary level of isobutyric acid than the HSD subtype (*P* = 0.047).

### Metabolite-associated biochemical pathways

**Fig. 7:**
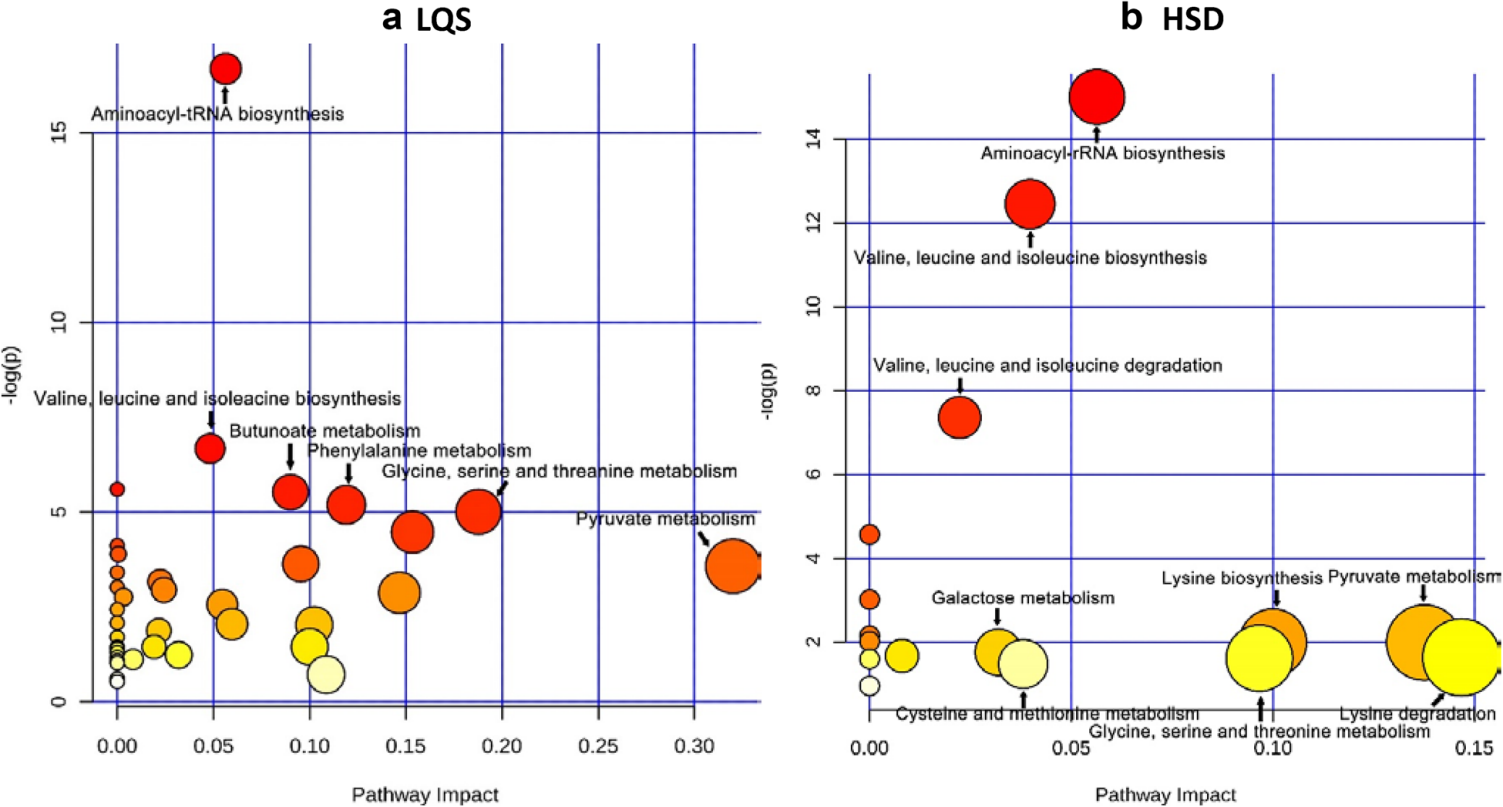
Biochemical pathway analysis shows biological impacts in differentiating Liver Qi Stagnation (LQS, **a**) and Heart and Spleen Deficiency (HSD, **b**) subtypes of MDD from healthy controls. X- and Y-axis indicates the magnitude of the impact and *p* value compared with healthy controls, respectively. Aminoacyl-tRNA biosynthesis and valine, leucine and isoleucine biosynthesis pathways are most closely associated with the two subtypes. The HSD subtype was additionally associated with valine, leucine and isoleucine degradation

The impact pathway analysis revealed that the differential metabolites of the LQS subtype were significantly associated with aminoacyl-tRNA biosynthesis; valine, leucine and isoleucine biosynthesis; butanoate metabolism; phenylalanine metabolism; and glycine, serine and threonine metabolism (Fig. 7a). The differential metabolites of the HSD subtype were heavily involved in aminoacyl-tRNA biosynthesis; valine, leucine and isoleucine biosynthesis; and valine, leucine and isoleucine degradation (Fig. 7b).

### The optimal metabolite profiles for separating TCM subtypes and healthy controls

**Fig. 8:**
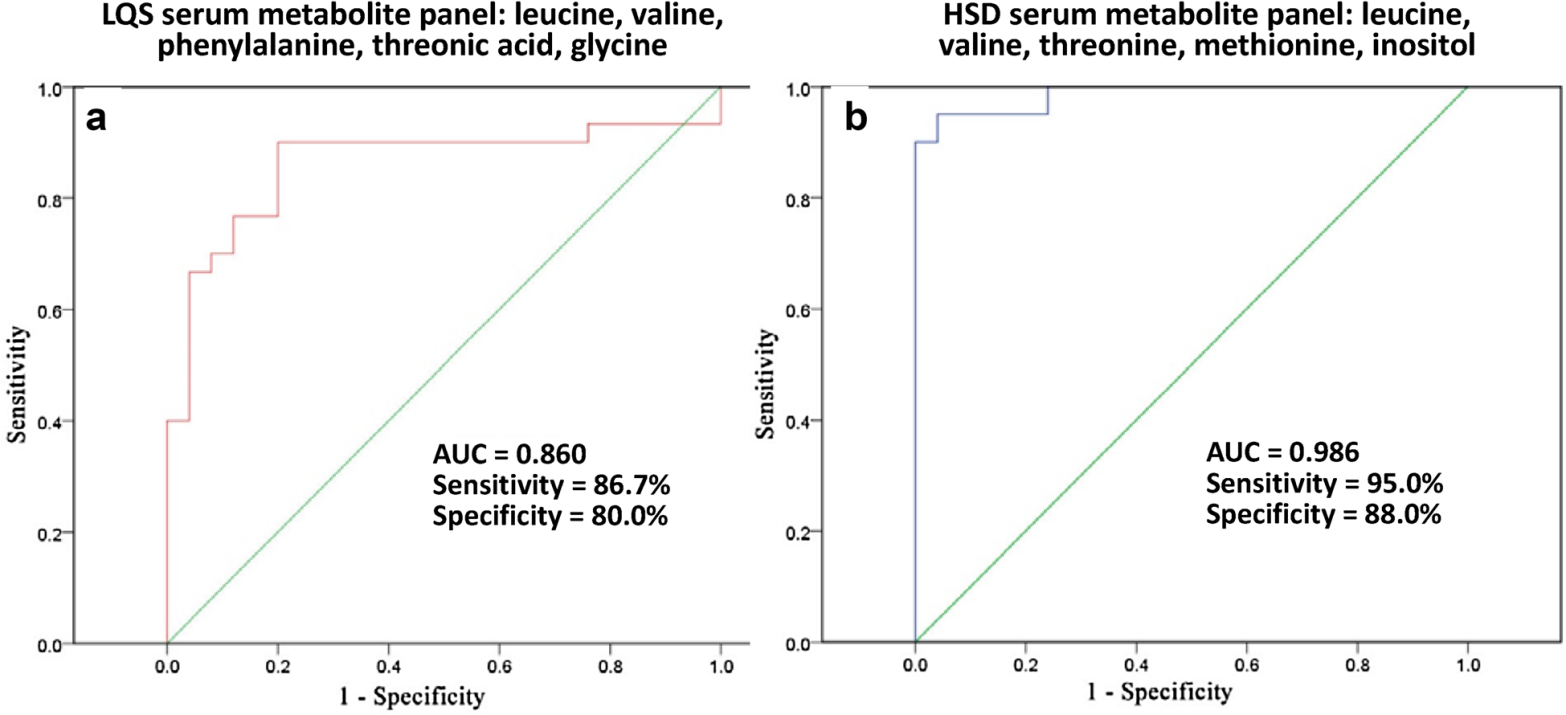
Receiver operating curve (ROC) analysis show the optimal serum metabolite panels that well differentiate Liver Qi Stagnation (LQS, **a**) and Heart and Spleen Deficiency (HSD, **b**) subtype from healthy controls

ROC analysis showed that the optimal metabolite profile with a combination of five serum metabolites (leucine, valine, phenylalanine, threonic acid, and glycine) well separated the LQS subtype and healthy controls with a sensitivity of 0.87 and a specificity of 0.80 (Fig. 8a). A combination of five serum metabolites (leucine, valine, threonine, methionine, and inositol) optimally dichotomized the HSD subtype and healthy controls with a sensitivity of 0.95 and a specificity of 0.88 (Fig. 8b). There were no optimal urinary metabolite profiles that could separate TCM subtypes and healthy controls at the acceptable sensitivity and specificity (data not shown).

## Discussion

This study represents the first attempt to introduce TCM-based diagnostic subtypes into the modern classification of depressive disorders. It not only could improve personalized treatment of depression, but also promote the integration of TCM theoretical concepts into the modern biomedical knowledge system. The sample recruited in this study was representative of MDD patients seen in China [7, 9, 11, 16].

The purpose of this study was to explore blood and urinary metabolomic evidence that could validate TCM diagnostic classification of MDD. A total of 28 meaningful metabolites were identified as potential biomarkers for differentiating MDD and healthy subjects. These metabolites mainly included amino acid metabolites involved in the synthesis of monoamine and amino acid neurotransmitters (l-tryptophan, phenylalanine, *o*-tyrosine, glycine, 3-hydroxybutric acid, and *N*-acetyl-l-aspartic acid), proteinogenic branched-chain amino acids (BCAAs) (valine, leucine, and isoleucine), oxaloacetate-derived amino acids (l-lysine, l-threonine and l-methionine) which are mainly involved in the biosynthesis of proteins and energy production via gluconeogenesis, and energy metabolism-related metabolites (l-lactic acid, palmitic acid, citric acid, stearic acid, pyruvic acid, and α-lactose) [24].

Clustering analyses revealed that the two TCM subtypes of MDD were well separated from healthy controls on serum metabolic profiles, but not on the urinary profiles. Likewise, ROC analysis displayed that the two optimal metabolite combination patterns for dichotomizing the TCM subtypes of MDD and healthy subjects were identified from serum samples, but not from urine samples. Similar results also have been observed in previous studies that attempted to identify blood and urine metabolic biomarkers for the severity of MDD [16, 20]. Blood metabolites seem to be more sensitive than urinary metabolites in distinguishing MDD from healthy subjects. This may be largely because blood metabolites can be maintained at a relatively constant state compared to urinary metabolites [25].

Although clustering analyses showed large overlaps in serum and urinary metabolite patterns between the two TCM subtypes of MDD, the LQS subtype had strong mixed correlations with multiple amino acid metabolites involved in the biosynthesis of monoamine and amino acid neurotransmitters, including phenylalanine, 3-hydroxybutric acid, *o*-tyrosine, glycine, l-tryptophan, and *N*-acetyl-l-aspartic acid; but the HSD subtype did not. ROC analysis further indicated that phenylalanine and glycine were among the optimal metabolite combination pattern for discriminating the LQS subtype and healthy controls. A preliminary study also has observed differences in multiple brain monoamine neurotransmission among TCM subtypes of MDD [26]. According to the presence and absence of stress prior to its onset, MDD can be classified as reactive and endogenous (melancholic) subtypes [2]. Stress-associated depression has been suggested to be more closely associated with dysfunction of brain catecholamine and amino acid neurotransmitters, in particular norepinephrine, glutamate and γ-aminobutyric acid systems [27, 28]. Clinically, the LQS subtype patients often exhibit stress-related comorbid symptoms and signs, such as nervousness, irritability, agitation, and a wiry pulse [2, 10]. It thus appears that the LQS subtype may represent a subpopulation of MDD characterized by abnormal biosynthesis of monoamine and amino acid neurotransmitters and closer associations with stress-related pathophysiology.

Moreover, both subtypes had opposing correlations with serum threonic acid, serum/urine l-methionine, and urine stearic acid. Serum threonic acid and l-methionine were also among the optimal metabolite combination patterns for respectively discriminating the LQS and HSD subtype from healthy controls. Threonic acid is a metabolite of ascorbic acid (vitamin C) and methionine is an essential amino acid in humans [24]. Dietary treatment with threonic acid- and methionine-derived supplements (e.g., magnesium-L-threonate (MgT) and S-adenosyl-methionine (SAMe)) had the benefits in improving multiple brain functions, in particular memory, depression, and anxiety [29–31]. Stearic acid is a saturated fatty acid which plasma concentrations have been found to be abnormally altered in patients with winter episode of MDD [32] and in the superior temporal gyrus and orbitofrontal cortex of bipolar patients [33, 34]. We further found that the LQS subtype had a markedly lower urinary level of isobutyric acid, a short-chain fatty acid than the HSD subtype. Plasma level of β-amino-isobutyric acid of autistic children has been shown to be significantly higher than that of healthy children [35]. These results suggest that threonic acid, methionine, stearic acid, and isobutyric acid are differentially associated with the two TCM subtypes and may be promising biomarkers for TCM classification of MDD.

However, the present study revealed that both subtypes had large overlaps in serum and urinary metabolite panels and shared several similar profiles, in particular proteinogenic BCAAs (valine and leucine) and energy metabolism-related metabolites (α-lactose and l-lactic acid), manifesting as consistent positive correlations with serum levels of valine and leucine, and negative correlations with levels of urine α-lactose and serum l-lactic acid. Biochemical pathway analysis displayed that both subtypes had consistent and closer links with valine, leucine, and isoleucine biosynthesis and aminoacyl-tRNA biosynthesis. ROC analysis also revealed that valine and leucine were involved in the two optimal metabolite combination patterns identified for discriminating the two subtypes and healthy controls. BCAAs and energy metabolism dysfunction is related to the pathogenesis of multiple psychiatric disorders and a common comorbidity in depressive disorders [36, 37]. The BCAAs play a key role in maintaining the supply of the neurotransmitters glutamate and γ-aminobutyric acid (GABA) [38]. Our study suggests that aberrant BCAA and energy metabolism seems to be distinct biochemical profiles in the pathophysiology of MDD and the BCAA-glutamate/GABA metabolic cycle in the brain may be potential biomarkers and therapeutic targets for MDD [36, 38, 39].

Several limitations of this study should be noted. First, there are 12 subtypes according to TCM classification of MDD [7–11], but we only examined the two most common and opposing subtypes of MDD. Whether other subtypes also have differential profiles needs further investigation. Second, the TCM diagnostic procedure was conducted mainly based on empirical practice and subjective assessments. Objective and precise TCM diagnostic approaches should be sought. Third, the sample size was relatively small with medication-free participant and gender imbalance. This may limit the applicability of the findings of this study. Whether similar results could be achieved in first-episode and treatment-resistant depressed populations needs further determination. Finally, we did not examine the differential response of the two subtypes to antidepressant treatment. Whether metabolomic biomarkers identified are effective in predicting TCM subtype-specific treatment outcomes deserves further examination.

## Conclusions

While the two subtypes are associated with aberrant BCAA and energy metabolism, the LQS subtype may represent a subpopulation of MDD characterized by abnormal biosynthesis of monoamine and amino acid neurotransmitters and closer associations with stress-related pathophysiology. Threonic acid, methionine, stearic acid, and isobutyric acid are differentially associated with the two subtypes and may be promising biomarkers for TCM classification of MDD. This study suggests that the TCM diagnostic subtypes could serve a valid classification of MDD.

## Additional files

**Additional file 1.** IRB approval copy.

**Additional file 2.** Minimum Standards of Reporting Checklist.

## Abbreviations

ANOVA: analysis of variance
BCAAs: proteinogenic branched-chain amino acids
DSM-5: Statistical Manual of Mental Disorders, Fifth Edition
GABA: γ-aminobutyric acid
GC–MS: gas chromatography-mass spectrometry
HAMD-24: 24-item Hamilton Rating Scale for Depression
HSD: Heart and Spleen Deficiency
LQS: Liver Qi Stagnation
MDD: major depressive disorder
OPLS-DA: latent structures-discriminant analysis
PCA: principal component analysis
ROC: receiver operating characteristic
TCM: traditional Chinese medicine
VIP: variable importance in the projection
