# Supplementary material for: Blood and urinary metabolomic evidence validating traditional Chinese medicine diagnostic classification of major depressive disorder
Source: Chin Med. 2018 Oct 25;13:53. doi: 10.1186/s13020-018-0211-z (PMC6203264; doi:10.1186/s13020-018-0211-z)
Supplement: Supplementary file 1 — Additional file 1. IRB approval copy. [file 13020_2018_211_MOESM1_ESM.pdf]

浙江省立同德医院医学伦理审查批件

伦理审查批号：浙同德伦理审字第［ 2014 ］009 号

项目名称	重性抑郁症中医辨证分型的神经化学、代谢组学和神经影像学的鉴别研究				
项目类别	临床科研	临床分期	临床验证	剂型/型号	
申办者	浙江省立同德医院				
临床批件					
研究单位	浙江省立同德医院心身科	项目负责人	刘兰英		
审查时间	2014 年 3 月 11 日	会议地点	浙江省立同德医院 2 号楼 14 楼 2 号会议室		
审查方式	会议审查☑ 快速审查☐				

伦理审查内容

审查材料	☑研究方案<br>☑知情同意书<br>☐临床观察表<br>☐研究者手册<br>☐研究者资质:参见项目组研究人员履历表<br>☐其他
投票结果	应到委员人数 13 人，实到 12 人，其中：投票人数 12 人，回避人数 0 人<br>结果：同意 12 票；作必要修正后同意 0 票；作必要修正后重审 0 票；<br>不同意 0 票；终止或暂停已批准的试验 0 票
审查结论	☑同意 ☐作必要修正后同意<br>☐作必要修正后重审 ☐不同意<br>☐终止或暂停已批准的试验

审批意见：

根据投票结果，本委员会同意自审批之日起开展该项试验，要求研究者和申报者严格遵守中国（GCP）、自觉接受国家有关法律和法规约束，注意防范不良事件，保护受试者权益和安全。研究过程中注意事项：

1. 修改方案、知情同意书和招募材料等应报伦理委员会审查批准后执行；
2. 若自同意研究之日起，一年内尚未完成研究，应每年向本委员会提交年度/定期跟踪审查报告；
3. 试验过程中发生以下情况应及时报告；①发生任何严重不良事件请立即 24 小时内报告；②违反研究方案；③暂停/终止研究；
4. 研究完成后提交总结报告。

主任委员（签字）：[illegible]

日期：2014 年 3 月 11 日

本次伦理委员会出席会议的委员情况

伦理审查会议编号：2014-02			
伦理审查时间	2014 年 3 月 11 日		
伦理审查地点	浙江省立同德医院 2 号楼 14 楼 2 号会议室		
姓名	性别	专业基本情况	签名
柴可群	男	中医内科	[illegible]
冯　斌	男	精神科	[illegible]
陈勇毅	男	中医老年病	[illegible]
王乃信	男	精神科	[illegible]
李亚平	男	中医心血管	[illegible]
黄飞华	男	中医内科	[illegible]
裘维焰	男	中医	[illegible]
张　春	男	骨伤	[illegible]
谢培怡	女	心血管	[illegible]
蒋坤庭	男	社区代表	[illegible]
王凤扬	男	法律	[illegible]
徐东娥	女	护理	[illegible]
周国仁	男	社区代表	[illegible]
